# Meio ambiente e saúde pública: as epidemias em Portugal na Idade Moderna

**DOI:** 10.1590/S0104-59702024000100058

**Published:** 2024-11-04

**Authors:** Maria Marta Lobo de Araújo

**Affiliations:** i Professora, Instituto de Ciências Sociais/Universidade do Minho. Braga – Portugal martalobo@ics.uminho.pt

**Keywords:** Meio ambiente, Saúde pública, Epidemias, Portugal, Morte, Environment, Public health, Epidemics, Portugal, Death

## Abstract

A questão da saúde pública e das epidemias conheceu nos nossos dias novamente a ribalta, devido à recente epidemia vivida e que, embora mais enfraquecida, continua a ser objeto da nossa atenção. Procuramos, neste artigo, compreender a relação existente entre meio ambiente e saúde pública e, nesse âmbito, estudar as principais epidemias conhecidas em Portugal ao longo da Idade Moderna. Foram várias as epidemias que encontraram no Portugal moderno condições favoráveis à sua propagação. O meio ambiente favoreceu o alastramento da doença e potenciou a sua virulência num período em que a medicina era incapaz de fazer face à doença, disparando as taxas de mortalidade.

## Abstract (en)

The issue of public health and epidemics is again in the spotlight today because of the recent epidemic we have experienced, which, although weaker, continues to be the object of our attention. In this article, we attempt to understand the relationship between the environment and public health, within this context exploring the main epidemics known to have occurred in Portugal in the Modern Age. Conditions in Portugal at that time were favorable for the spread of several epidemics; the environment encouraged the spread of disease and boosted virulence at a time when medicine was unable to cope, causing mortality rates to soar.

Com lugar ganho na agenda da saúde mundial, a pandemia pelo SARS-CoV-2 experimentada de forma muito intensa recentemente, e com a qual continuamos a conviver, convoca o nosso olhar para o tempo longo da história e para nele repousar na Idade Moderna portuguesa, relacionando meio ambiente e saúde pública, com destaque particular para as principais pestes e epidemias. Mais do que contar mortos e demonstrar o impacto das doenças na sociedade, interessa-nos perceber a influência do meio ambiente na saúde das populações e de que modo ela se interligava com as epidemias. É, então, sob o prisma das condições sociais e ambientais que analisaremos as epidemias, relacionando-as com os contextos, procurando-se discutir condições de higiene, insalubridade, acesso aos bens alimentares essenciais, pobreza, fome, medo e morte.

Apesar dos efeitos muito negativos da citada doença, ela fez relançar os estudos sobre as epidemias, de que são exemplo múltiplas iniciativas científicas, como seminários e congressos, assim como livros, capítulos de livros e artigos recentemente publicados (Cueto, 2021). A investigação histórica sobre essa temática ganhou um novo impulso e permitiu alertar para as grandes diferenças existentes, se compararmos com a Idade Moderna, nomeadamente os avanços da medicina, as novas formas de enfrentar a doença, mas também pôs a nu os problemas estruturais, muitos deles decorrentes da organização das sociedades, assentes nas desigualdades, e a maneira como estavam integradas e se perspectivavam (Cueto, 2021).

Portugal conheceu, ao longo dos séculos XVI a XVIII, muitas pestes e epidemias, o que, aliás, não constituiu particularidade, porquanto todas as regiões europeias se confrontaram igualmente com elas, registando similitudes quer no tocante às ocorrências, quer à forma de as enfrentar.

O estudo das epidemias no Portugal moderno encontra eco em alguns trabalhos produzidos mais recentemente para algumas regiões, pese embora as dificuldades presentes para a elaboração de uma síntese, devido ao desconhecimento existente para muitas das suas partes. Acresce ainda o fato de os estudos conhecidos se cingirem, quase por inteiro, às cidades e às zonas de fronteira. Embora impossibilitados de traçar um panorama nacional das epidemias, podemos falar de uma “geografia da saúde por regiões” (Oliveira, 2015, p.597). Pese embora as dificuldades, para conhecer e analisar algumas epidemias, é possível acompanhar alguns itinerários, desenhando caminhos de passagem, de fome, de terror e de morte.

A situação geográfica portuguesa tornava o país vulnerável a surtos epidémicos, que entravam por mar (Barros, 2013) ou por terra (Abreu, 2018) e se espalhavam com muita facilidade em regiões mais ou menos alargadas, dependendo da forma como poderes públicos e privados encaravam a saúde pública e procuravam combater a doença, da atitude das populações, da capacidade que tinham para a enfrentar, bem como dos instrumentos disponíveis para o fazer. As relações comerciais, a circulação dos exércitos e os circuitos migrantes debilitavam as fronteiras portuguesas, que assistiram à entrada das doenças através dessas linhas, impondo temor e causando elevado número de mortes. Quase todas as epidemias e pestes que assolaram o Portugal moderno vieram da Europa ou do Norte de África. Antes de vencerem as nossas fronteiras tinham já causado grande mortalidade em outras regiões e, chegadas a Espanha, rapidamente se propagaram ao nosso país. O mar também facilitava o contágio, pelo que vindas de mais perto ou de mais longe, acabavam por se introduzir no nosso território.

E tal como na Europa, em Portugal a medicina não respondia aos problemas colocados, e a sociedade era incapaz de fazer estancar as epidemias. Perante este cenário, as epidemias não atacavam apenas indivíduos, mas eram experimentadas por toda a comunidade, desestruturando-a e colocando-a em permanente sobressalto.

## Meio ambiente e saúde pública

As doenças que se fizeram sentir ao longo dos tempos foram mudando. São tributárias da forma como viviam as populações, da sua cultura, da sua alimentação e do relacionamento que estabeleciam com o meio ambiente. Ao mesmo tempo, deixaram marcas na sociedade, na economia, na cultura e nas pessoas (Esteves, 2020).

Certos de que os pobres assumiram no palco das epidemias um dos principais papéis (Cueto, 2021), propomo-nos analisar a forma como o meio ambiente influenciava diretamente as suas vidas e como a complexidade das sociedades os tornava um dos alvos preferenciais. Embora a pobreza não seja causadora diretamente de doenças, ela pode potenciá-las, quando associadas ao meio ambiente e às carências alimentares, bem como de higiene, principalmente nos centros urbanos, devido aos aglomerados populacionais (Mckeown, 1990). Era por essas razões que algumas pessoas resistiam melhor à doença do que outras, sobretudo quando o sistema imunológico se encontrava mais robusto, respondendo de forma mais eficaz à infeção, como era, por exemplo, o caso da tuberculose (Hays, 2009).

A Época Moderna portuguesa conheceu, como a Europa, várias alterações climatológicas, bem analisadas no recente trabalho de Luís Silva. O autor estuda de forma minuciosa as mudanças climáticas no Noroeste de Portugal entre o século XVII e meados do século XIX, fazendo também várias incursões em todo o território nacional, demonstrando a forma como eram vistas pela população e os meios por esta usados para aplacar a considerada ira divina. Por meio das chamadas “procissões pelo tempo” – *Pro serenitate* e *Pro Plubia* –, o autor estabelece a cronologia dessas manifestações religiosas levadas a cabo pelas populações, quando o tempo não as favorecia. Assim, chuvas excessivas e prolongadas, por vezes, acompanhadas de quedas de granizo, ventos fortes e tempestades, tinham efeitos nos rios, provocando cheias, sobretudo no inverno, atrasando as sementeiras, quando não as prejudicavam também. Igualmente, períodos de seca prolongados eram prejudiciais para as culturas e punham as populações em risco. Sempre que as chuvas eram abundantes, como aconteceu no Porto, em 1739, que se prolongaram durante alguns meses, fizeram transbordar as margens do rio Douro, ocasionando cheias e prejuízos às populações (Amorim, Silva, Garcia, 2017). Sentiam-se os efeitos não somente em terra, mas também nos rios e no mar. Os rigores meteorológicos tiveram impacto nas crises alimentares e causavam destruição patrimonial, mortes humanas e empobrecimento (Silva, 2019).

Quando a chuva era acompanhada de ventos, neve e trovoada, acabava com todos os produtos agrícolas da estação, provocando prejuízos avultados. O inverno de 1711 registou muito frio, deixando de existir nos campos alimento para o gado, e as sementeiras não estavam garantidas no Sul do país, o que potenciou o surgimento de doenças na capital (Silva, 2019). O mesmo clima também foi sentido no Norte, onde chuva intensa marcou os dias da população.

Em casos de secas prolongadas, a agricultura também sofria, perdendo-se as sementeiras e frutos, o que causava esterilidade. Falta de pasto, falta de água e perda de gados eram fatores que punham em perigo o parco equilíbrio das populações rurais. Também chuvas fora de tempo constituíam motivo de preocupação pela incerteza respeitante às colheitas. As duas primeiras décadas do século XVIII foram muito instáveis em termos climatológicos, registando-se chuvas no Minho durante o verão.

Entre 1768 e 1770 todo o Minho conheceu intensa chuva, geadas e neves nos outonos e invernos, bem como carestia e muita pobreza. O prolongamento das neves e das chuvas destruiu toda a colheita e alastrou a fome na região, muito particularmente em Braga. O resultado foi uma forte epidemia sentida em várias regiões minhotas e particularmente na sua capital, onde o tifo matou cerca de 12% da população. Perante as más colheitas, o preço dos cereais ficou muito elevado e inacessível às populações pobres. Os cronistas fazem eco de mulheres e crianças gritando pelas ruas com fome (Araújo, Miguel, 2020) e das medidas tomadas pelo arcebispo e pela Câmara para abastecer as populações de pão (Oliveira, 1996, p.253-256; Silva, 2019; Araújo, Maria, 2020, p.186). Era uma situação de fome extrema, conhecida também em várias regiões europeias, dando origem a motins de fome, à doença e à morte. Em Braga, apesar das diligências efetuadas, foi inevitável o medo, o sofrimento e a morte. Corpos debilitados, por carência alimentar, estavam mais suscetíveis ao contágio, por mais facilmente contraírem a infeção e dificilmente a combaterem. A morte era inevitável.

**Figura 1:**
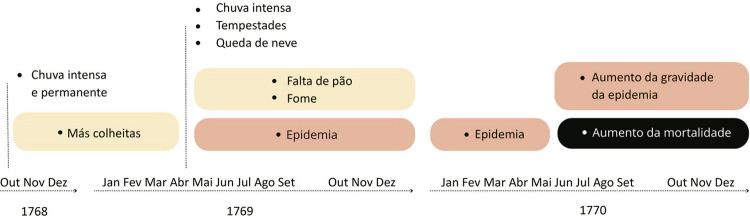
: Meio ambiente e saúde pública em Braga, 1768-1770 (Araújo, Miguel, 2020)

A análise do mapa segue o relato do cronista Miguel Luís Araújo e demonstra a sequência estabelecida entre o clima e os maus anos agrícolas, a epidemia e a morte em Braga. O hospital da cidade ficou sobrelotado quando surgiram as “febres malignas e contagiosas”, que deixaram a “gente pasmada”. Incapaz de compreender o sucedido, a população refugiava-se na crença e aguardava que as preces fossem suficientes para agradar ao divino. Recebiam-se doentes em todos os espaços, mesmo que fossem corredores e varandas, montaram-se estruturas de cura noutros locais, deixando o hospital para internar o sexo feminino e enviando o masculino para o extinto colégio dos jesuítas, após pequenas alterações para o efeito. O temor da doença era tão grande que nem os profissionais de saúde queriam tratar os internados, sendo necessário contratar outros assalariados. Eram tempos de luta contra a fome e a doença, estava instalado o pânico, e o hospital tinha-se transformado num lugar de morte.

Essa epidemia foi também registada noutras regiões: Viana do Castelo, Ponte de Lima, Beira e Trás-os-Montes conheceram igualmente os seus efeitos (Araújo, Miguel, 2020).

Considerada a emergência da situação, quando surgiam epidemias e os hospitais existentes não conseguiam responder e atender todos os que os procuravam, erguiam-se outros, por vezes, temporariamente, e tomavam-se medidas com vista a melhorar as condições de salubridade (Vigarello, 2001). A estrutura hospitalar portuguesa era, na Idade Moderna, formada maioritariamente por pequenas unidades, permanentemente lotadas e com problemas vários. Destinados aos pobres, que neles eram tratados gratuitamente, os hospitais estavam na sua maioria sob administração das Misericórdias e conheciam muitos constrangimentos. A sua dimensão era apenas a face mais visível, porquanto problemas de higiene, de salubridade, de falta de cuidadores, de roupa e até de leitos expunham políticas de assistência, mas igualmente as muitas carências sentidas dentro de portas. Sempre que podiam, essas confrarias foram promovendo o surgimento de novos complexos, como aconteceu no Porto com o hospital de Santo António e em Viseu, com a substituição do hospital das Chagas, no século XVIII, por um novo, enquanto outras mantiveram as estruturas existentes, sujeitando-as a obras de melhoramento e aumento, como se verificou em Braga e em Viana do Castelo, também em setecentos, entre outros. A reduzida capacidade de internamento era talvez a mais penalizadora em tempo de epidemia, porquanto não só não se abria a porta a todos os que necessitavam, como o contágio constituía um grande problema. Os hospitais das Misericórdias estavam impedidos de receber enfermos com males contagiosos, a não ser os que reuniam condições para prestar serviços adequados, como acontecia em alguns de maior dimensão, no caso dos portadores de sífilis. Esses tratamentos efetuavam-se apenas em duas ocasiões ao ano e de forma muito controlada. Os enfermos eram internados em enfermarias separadas dos restantes, normalmente situadas em pisos diferentes, estavam sujeitos a cuidados especiais, muito particularmente no que se referia à roupa, ao calçado e ao tratamento. Todavia, quando rebentava uma epidemia, as estruturas hospitalares acabavam por se vergar à pressão da doença e recebiam os seus portadores, o que normalmente originava o caos em todos os setores.

Aquando da epidemia anteriormente referida para a cidade de Braga, procissões, preces, ladainhas e novenas foram feitas, quer por todo o aparelho religioso da cidade, quer pela sua população. A ordem para reunir e rezar vinha do arcebispo e todos a acatavam, por entenderem ser o castigo de origem divina (Araújo, Miguel, 2020).

A última década de setecentos ficou também ela marcada por anos de frio intenso e por um conjunto de epidemias já conhecidas para o Norte e Sul de Portugal (Silva, 2019; Abreu, 1997). As epidemias iam e vinham, adotando ritmos variados, mas mantinham uma presença assídua junto das populações. As fontes não apontam uma doença específica, falam de “febres malignas” ou de “doenças malignas”, que duraram quase toda a década.

## A insalubridade da água

Portugal, na Idade Moderna, à semelhança de muitos outros, era um país com muitos fatores de risco, que potenciavam a doença e a sua propagação. A água a que a maioria da população tinha acesso não reunia condições de ser consumida, tonando-se causadora de muitas doenças, entre as quais se podem destacar a febre tifoide, a diarreia, a disenteria e a cólera. Águas estagnadas eram ainda locais favoráveis para o surgimento de mosquitos, que podiam provocar febre amarela e malária, esta última com maior ocorrência no verão, quando o mosquito se movimenta mais (Watts, 1997), por isso deviam ser fervidas antes de ingeridas. Alguns espaços constituíam locais de contágio, como eram os charcos, os pântanos, as águas paradas e os rios. Assim mesmo, não se devia beber água dos rios, embora fosse gratuita, por poder estar conspurcada. Como a grande maioria da população não dispunha de água canalizada em sua casa, ia buscá-la às fontes, sendo necessário abastecer todos os dias, por falta de locais de armazenamento. Podia também colhê-la em poços, mas nem sempre era sinónimo de qualidade, porquanto podia estar sujeita a infiltrações de águas sujas. A melhor era a que era paga, trazida pelos aguadeiros às moradias mais ricas e de posses, mas mesmo essa, nem sempre reunia condições de salubridade, pois a sua qualidade dependia dos locais de proveniência. Todavia, a maioria da população não dispunha de condições financeiras para a fazer chegar à sua casa.

De um modo geral, as cidades eram mal abastecidas de água, como se comprova para Lisboa. Apesar dos canos, chafarizes e até aquedutos, a maioria da população da cidade não tinha acesso a água potável (Alho, 2021).

Mas se a água era um fator relevante, também o ar que se respirava era muito considerado, tal como a tranquilidade, o descanso, o resguardo do corpo no inverno e a proteção contra as correntes de ar. O ar facilitava a transmissão de doenças, como acontecia com a gripe, a tuberculose, a varíola, o sarampo e a difteria. Alguns insetos eram igualmente portadores de doenças, como os piolhos, as pulgas, os mosquitos, encarregando-se de disseminar doenças como o tifo, a febre amarela e a malária, encontrando-se amiúde entre os seres humanos (Carmona García, 2021; Mckeown, 1990).

## A alimentação

Uma parte substancial da população portuguesa moderna tinha o pão como base alimentar, pois a desigualdade social mantinha-se também à mesa, todavia, esse alimento conhecia muitas variedades, dependendo da farinha que era usada para o confeccionar. De acordo com a farinha, os diversos tipos de pão conheciam preços diferentes, e o melhor não era acessível aos camponeses nem aos grupos sociais mais desfavorecidos, que se viam obrigados a ingerir o pão de pior qualidade. Os melhores cereais que originavam pão de qualidade eram acessíveis apenas a alguns, ou seja, aos ricos. Os pobres consumiam o pão mais barato, logo, de qualidade inferior (Carmona García, 2021, p.27).

Portugal nem sempre conseguia ter pão suficiente para abastecer a população, vendo-se obrigado, em vários momentos, a importar cereais de vários pontos da Europa e mesmo do Norte de África. Mas este nem sempre chegava nas melhores condições, podendo ser nocivo à saúde. Como a base alimentar era o pão, em tempos de crise, rompia-se o precário equilíbrio alimentar, conhecendo-se dificuldades acrescidas, quando não mesmo a fome. Associada à feitura e venda de pão, está também a falta de limpeza das padeiras, o que preocupava as autoridades locais, fazendo, por vezes, alusão a esse problema nas posturas municipais (Araújo, 2022).

Todos os alimentos saídos da agricultura estavam muito dependentes das condições meteorológicas, pelo que em anos adversos a produção diminuía, os preços dos cereais subiam, a população mais vulnerável não podia aceder ao pão, sofrendo de subnutrição. Esta era uma das consequências mais gravosas, mas ficava também a nu a falta de eficácia da rede abastecedora de produtos. Em pouco tempo, os preços podiam duplicar ou quadruplicar, provocando grandes constrangimentos às famílias. Porém, não raras vezes, não se elevava apenas o preço dos grãos, também outros produtos conheciam uma escalada de preços, o que agravava a vida das populações. O vinho e o sal subiram também de preço em Braga, quando a cidade conheceu uma forte epidemia entre 1712 e 1713 (Tedim, 1764, p.2).

Sempre que faltava o pão, recorria-se ao que estava à mão, normalmente produtos de qualidade inferior, provocando debilidade do corpo, com repercussões mentais. Não era tanto a falta de comida, mas o estado de subnutrição em que as pessoas se encontravam (Carmona García, 2021, p.42). A alimentação dos grupos sociais mais vulneráveis raramente incluía carne, bem como peixe fresco, o que tornava as refeições pouco variadas e carentes de proteínas, vitaminas e minerais. Com fome, os corpos ficavam mais vulneráveis à doença, contraindo-a mais facilmente e resistindo com mais dificuldades. Tocados pela doença e se o organismo não respondia, engrossavam as já elevadas taxas de mortalidade.

Perante a falta de alimentação, a população pobre movimentava-se principalmente em direção às cidades, procurando meios de subsistência, o que nem sempre era possível encontrar, devido à crise. Durante a grande peste de 1569, Lisboa recebeu muitas vagas de pobres, idos de várias regiões, que vagueavam pelas ruas sem acesso a comida e teto. A peste bubónica matou na cidade vários milhares de pessoas (Rodrigues, 1990), pese embora as medidas de assistência implementadas pela Coroa, principalmente para os grupos desenraizados e mais vulneráveis. Essa doença continuou a matar nos anos seguintes, havendo rebates de peste em várias regiões portuguesas (Lemos, 1991). A capital foi amiudadamente fustigada por essa peste, sujeita que estava à chegada de gente de variados reinos e lugares, o que a fragilizava em termos terrestres, mas principalmente marítimos, justificando a presença de “dispositivos sanitários” para quem a procurava (Abreu, 2020, p.96-98).

Entre 1579 e 1582, Portugal conheceu provavelmente mais um surto pneumónico, pese embora outras doenças possam ter estado associadas, como o tifo, a malária e a varíola. Coincidente com o seu início, a batalha de Alcântara (1579) implicou a movimentação de militares portugueses e castelhanos, partidários de dom António, prior do Crato, e de dom Filipe II de Espanha, gerando violência e mortes.

**Figura 2:**
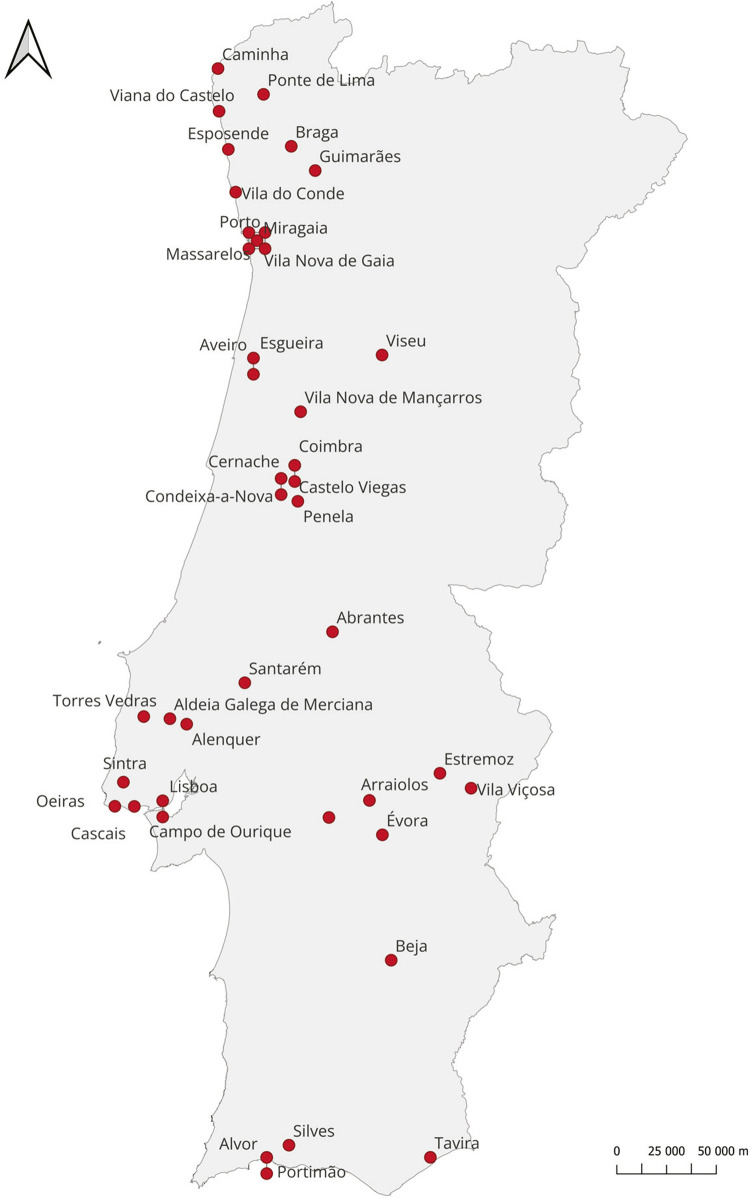
: Mapa da circulação da peste, 1579-1582 (Oliveira, 2015; Lemos, 1991; Rodrigues, 1993)

O mapa ilustrado na Figura 2 apresenta os locais que enfrentaram peste entre 1579 e 1582. A sua leitura deve ser efetuada com cuidado, pois os lugares apontados não se referem a todos onde houve peste, mas somente aos que são conhecidos. Também não indicam a mortalidade atingida em cada localidade, por não termos essa informação, pelo que desconhecemos a intensidade atingida em cada região. Os estudos consultados não mencionam todas as regiões, como acontece com Trás-os-Montes e as Beiras, pelo que não figuram com casos de peste, embora seja dúbio que não a tenham conhecido nesse período. Ignoramos ainda a correspondência entre o tempo cronológico e a epidemia em cada localidade. Embora os estudos refiram amiudadamente que todo o Minho foi infetado, não dispomos de informações que o comprovem, pelo que apenas mencionamos as localidades que neles são referidas. Apesar das limitações, fica patente a capacidade de transmissão e a circulação da epidemia, não somente ao longo de quatro anos, mas também em muitas regiões portuguesas.

Em 1580, a peste impediu que as cortes de aclamação de Filipe II se reunissem em Lisboa, sendo Tomar o local escolhido para o efeito. A peste regressou no ano seguinte, quando a falta de alimentos fez disparar o preço dos produtos, para novamente se conhecer uma situação de maior acalmia, até surgir um novo surto em 1582, dessa vez de menor gravidade.

Nos últimos anos de Quinhentos uma nova peste atacou várias regiões de Portugal, causada por surtos de cólera, varíola e sífilis. Toda a década de 1590 conheceu anos de epidemias, motivando grande instabilidade e mortes. Em 1599, Lisboa registou elevada mortalidade, tal como Guimarães, Esposende, Coimbra, Arrifana e o Minho (Barros, 2013, p.170-171). Vinda da Galiza, a peste bubónica fez-se sentir na capital e dela passou a outras regiões, estendendo-se rapidamente. Os pobres que vagueavam pelas cidades, com incidência particular em Lisboa, eram acusados de espalharem a doença e de consumirem o pouco alimento existente. Durou vários anos, vindo e indo até ser debelada (Rodrigues, 1990). Altamente contagiosa, a doença era conhecida pelos bobões surgidos em diversas partes do corpo dos infetados, causadores de um cheiro considerado intolerável por alguns sobreviventes (Snowden, 2020). Em 1645, depois de ter assolado vários contextos europeus, bateu à porta de Tavira e daí galgou para outros locais algarvios, como Lagos, Silves, Vilamoura, Albufeira, Alcantarilha, Ameixilhoeira, Estombar e Alagoa, tendo quatro anos mais tarde surgido associada à pneumónica. Essas duas doenças atingiam uma taxa de mortalidade elevadíssima. A peste bubónica sentiu-se igualmente no Alentejo, onde se espalhou por várias regiões na mesma altura, tendo obrigado a cidade de Évora a tomar medidas severas de proteção (Gusmão, 1969), todavia, como refere António de Oliveira (2015, p.616), no século XVII, as doenças que mais afetaram Portugal foram “as sezões, as doenças eruptivas, os tifos, a sífilis, a tuberculose e a varíola”.

Famélicos, sem hábitos de higiene e com todas as necessidades, os grupos de pobres constituíam um risco para as cidades e tornaram-se indesejados por serem considerados focos difusores de doença. Mas não eram apenas os forasteiros que criavam problemas de insalubridade, os próprios habitantes não tinham hábitos de higiene corporal, nem pública, sendo, portanto, necessário impor um conjunto de medidas. Depois de instalada a doença, ou em vias de bater à porta, o poder local entrava em ação, tomando medidas para a conter ou eliminar. A nomeação de um guarda-mor da saúde, o encerramento das cidades, por meio de portas fechadas, instalação de quarentenas, cordões sanitários e lazaretos foram algumas das medidas implementadas (Abreu, 2018).

Dependendo da sua virulência, das medidas vindas do poder central, que o poder local aplicava (Abreu, 2006), e da capacidade de os corpos resistirem às epidemias, estas podiam ser mais ou menos prolongadas e a mortalidade ser mais ou menos expressiva.

## A falta de higiene corporal

A falta de higiene constituía também um grave problema, quer em termos particulares, quer públicos. A não lavagem do corpo, como se aconselhava, por se entender que a água podia causar a doença, levou as pessoas a higienizar somente as partes descobertas, ou seja, as mãos e a cara. Acreditava-se que a água contribuía para debilitar o corpo e expô-lo a perigos, pelo fato de a dilatação dos poros contribuir para perda de força vital (Carmona García, 2021, p.77). A denominada “limpeza seca” sobreviveu durante séculos, sob o aconselhamento de apenas se substituir a camisa, peça de roupa mais próxima da pele, que absorveria as secreções (Vigarello, 1988). Era usada por ambos os sexos, diferia de acordo com o estrato social e, no começo da Idade Moderna, podia também ser usada para dormir (Sequeira, 2023, p.707). Assim, mudar a camisa era significado de estar limpo, o que implicava ter mais do que uma. Para ser lavada, era necessário que existisse uma substituta, e nos grupos mais desfavorecidos ela não só não existia, como as mantas que eram usadas na cama serviam também para resguardar o corpo durante o dia. Era assim que muitos pobres pediam pelas ruas, com as mantas às costas. Equacionar, portanto, a mudança de camisa para as camadas mais desfavorecidas da população era uma miragem que apenas acontecia de forma muito espaçada. Fazer a cama e mudar a roupa era também muito variável nos hospitais, dependendo de cada caso, e estava diretamente relacionado com a capacidade financeira da instituição para comprar roupa. Quase sempre usada e velha, ia sendo renovada lentamente, de acordo com as necessidades e políticas de investimento de cada instituição. Sem grande disponibilidade para proceder a mudas de roupa, a que saía para lavar devia regressar branca, dentro do prazo estabelecido, o que, por vezes, originava pressão junto das lavadeiras.

Quando aceites nos hospitais, os pobres, antes de serem internados, tomavam banho, bem como lavavam as mãos antes das refeições, quando lhes era servida água para o efeito, por meio de um jarro. Em algumas dessas instituições de saúde, usavam também guardanapos para se limparem, aquando das refeições. Apesar da preocupação com a higiene dos internados, no hospital de São Marcos, de Braga, constatou-se, em 1740, a existência de parasitas no corpo de um enfermo que acabava de morrer, assim como roupa apodrecida no corpo de outros doentes (Araújo, 2014). Esses parasitas podiam circular não somente pela pele, mas também no cabelo e atacavam pobres e ricos. A sua presença espalhava a doença, podendo provocar infeções cutâneas, como a sarna (Lindemann, 2002). Mas não eram só os corpos que precisavam de ser limpos; também os colchões e as camas necessitavam de atenção particular. Se a palha não fosse periodicamente removida e substituída por outra, podia potenciar o surgimento de insetos nocivos à saúde pública (Carmona García, 2000). O mesmo processo deveria ser feito, aliás, para a roupa exterior. A roupa era cara na Idade Moderna e, no caso dos pobres, ela era geralmente obtida por meio de dádivas, quer de particulares, quer de instituições de assistência.

A localização dos institutos de saúde podia também constituir um problema. Os hospitais estavam quase sempre situados no centro das cidades e vilas, o que podia ser grave em tempo de epidemias. Essas instituições procuravam proteger-se para evitar o contágio dentro de portas, pugnando-se para remover as imundices e esterco das ruas circundantes, de forma a mantê-las limpas, ao mesmo tempo que faziam temer as populações vizinhas quando tratavam os infetados, por estes recearem que a doença galgasse os muros hospitalares.

Uma população mal alimentada, malvestida, maioritariamente a viver em habitações sem condições, e sem hábitos de higiene estava mais vulnerável às enfermidades, contraindo-as e tornando-se sua transmissora. A falta de higiene corporal e da roupa facilitava o surgimento de pulgas, percevejos e piolhos, insetos difusores de doenças. O piolho tornou-se responsável pela transmissão de algumas epidemias, nomeadamente de tifo (Ujvari, 2014). Ora, o tifo era “companheiro da fome” (Oliveira, 2015, p.629), de modo que ameaçava as populações sempre que existia uma crise alimentar, ou outra situação crítica, como se verificou em período de guerras, nomeadamente na da Restauração (1641-1668). Em 1658, durante o cerco de Elvas, rebentou um surto de tifo que se espalhou para outras regiões alentejanas, como se verificou em Lisboa, em Vila Viçosa, no Alentejo, e em Ponte de Lima, no Minho (Rodrigues, 1990).

Depois das décadas de 1620 e 1630 serem muito duras para os portugueses, devido principalmente à escassez de produtos e às altas taxas de impostos, as seguintes caracterizaram-se não somente pela presença de muitas doenças, mas igualmente pelo referido conflito bélico. O tifo surgiu no Sul de Portugal, associado à peste bubónica (Oliveira, 2015) e ainda à varíola, à difteria e à malária. Doença de grande gravidade, sabe-se que viver após contágio ou morrer dependia do estado de saúde do contagiado, ou seja, do seu sistema imunológico e da sua idade. Tendo mais incidência a partir do século XVII, e com prazo de incubação de uma a três semanas, a doença atacava preferencialmente os desnutridos, sobretudo no inverno e primavera, não raras vezes associada a outras doenças do foro respiratório. Nesse arco temporal, o piolho encontrava melhores condições para se multiplicar, devido às camadas de roupa que se punham no corpo e à falta de higiene deste.

Vinda por mar ou por terra, a doença encontrava terreno fértil para se propagar, pese embora a existência de regimentos que foram surgindo, para a capital e para o reino, para a suster e combater (Canas, 2021; Gusmão, 1969). Medidas destinadas aos enterramentos, como efetuá-los à noite, fora das igrejas, e covas com mais de dois palmos de profundidade, para que os fluídos não corrompessem o ar, procuravam afastar os empestados dos sãos. Estes instrumentos regulamentares indicavam-se ainda algumas medidas cautelares, relativas à alimentação, ao vestuário e às habitações (Rodrigues, 1993).

Em termos públicos, uma das preocupações das autoridades estava associada aos locais de venda de produtos alimentares. Como vimos, as padarias eram tidas em consideração, mas também os açougues, lugares onde, não raras vezes, as carnes estavam adulteradas, sendo mesmo assim vendidas, colocando em risco a saúde pública. Também os chafarizes, fontes e canos de condução de água eram tidos em consideração e mantidos sob vigilância, impedindo-se a população de lavar as entranhas dos animais nesses lugares e de deitar lixo e animais mortos para eles. Proibia-se ainda a lavagem de roupa nos chafarizes públicos, impedindo que a água ficasse conspurcada e prejudicasse a saúde da população. Desses locais era colhida para as pessoas beberem, mas por lá circulavam animais, como cães, gatos e outros, sujando e prejudicando a saúde das pessoas. O mesmo pode ser dito sobre alguns rios, para onde se despejava muita sujidade.

## Ruas e praças

Água inquinada, fontes sujas, esgotos a céu aberto, lixo aos montes nas ruas e nas praças faziam parte das cidades europeias da Idade Moderna. As ruas e as praças públicas amontoavam lixo e sujidade, tornando-se espaços potencialmente perigosos. Animais domésticos, como cães e gatos, passeavam livremente por ruas, praças, vielas, becos e travessas, tal como suínos, gado vacum e cavalar.

Parte do abastecimento das cidades era efetuado por meio de carros puxados por vacas ou mulas, o que as tornava sujas, com dejetos, e irregulares. O fato de muitas artérias serem em terra batida, o que vai sendo alterado nos séculos XVII e XVIII em algumas cidades, não contribuía para maior limpeza. Para além de animais, as ruas e as vielas tinham ainda lixo doméstico e resultante de atividades profissionais. A existência de vestígios de atividades profissionais, como acontecia no caso dos curtumes e dos açougues, criava verdadeiras lixeiras, originando maus cheiros. A população entendia a rua como o prolongamento da casa, servindo-se desse espaço para depósito de imundices, quer fossem sólidas, quer em estado líquido. Vários desses locais serviam igualmente de pouso aos pobres, alguns deles portadores de animais. Acresce ainda a falta de arejamento de algumas delas, por serem estreitas e tortuosas. Uma outra característica das ruas e praças prende-se com os odores existentes, o que constituía uma ameaça permanente: a presença de animais em putrefação, as bancas de venda de produtos, como peixe e carne, o lançamento de águas e dejetos domésticos para a via pública, por não existir saneamento, a escassez de entrada de luz em algumas vielas e até a humidade de várias originavam maus cheiros e a presença de insetos, e em nada contribuía para a preservação do ambiente, da saúde e do bem-estar da população. O ar estava corrompido, como se acreditava, o que potenciava a circulação dos miasmas e a transmissão das doenças. A crença na corrupção do ar fazia aumentar o temor da população, por poder disseminar a doença por meio do contágio. Esse medo dizia respeito quer aos vivos infetados, quer aos mortos, falecidos por peste. Era por essa razão que em período epidémicos as autoridades públicas ordenavam a queimadura de ervas cheirosas em certos locais, dando prioridade às ruas e praças, para criar bons ares, bem como a construção de cemitérios mais afastados das habitações e a feitura de sepulturas mais fundas para evitar que os fluídos atingissem os sãos. Os cemitérios contribuíam para a insalubridade urbana devido às sepulturas pouco profundas e com ossadas a descoberto, o que potenciava a proximidade de animais e a contaminação da água. Nas enfermarias dos hospitais colocava-se alecrim e em algumas delas a hospitaleira circulava com brasas antes do médico e do cirurgião visitarem os internados.

Respirar bons ares estava em relação direta com os cuidados em ingerir alimentos e bebidas (Barreiros, 2014). Ambientes sobrecarregados, sem arejamento e sem iluminação natural eram tidos como insalubres, infetos e potenciadores de doenças.

Principalmente no inverno, mas sempre que chovia as ruas enchiam-se de charcos, tornando-se lamacentas e com muita sujidade, o que constituía uma ameaça para a população. Essa situação facilitava o surgimento de animais e insetos perigosos por potenciarem a proliferação de doenças, o que era desconhecido das pessoas. Não somente ratos, mas também cães, pulgas e piolhos eram presença constante nas ruas das cidades e também nas habitações. Era por essas razões que, em tempo de epidemia, as cidades se tornavam mais perigosas que o campo e quem podia fugia delas o mais depressa possível, mesmo tratando-se de comunidades religiosas obrigadas à clausura (Rodrigues, 1993; Silva, 2011), indo para lugares considerados mais seguros. Os que ficavam, fechavam-se em suas casas, limitando ao máximo o contacto com o exterior.

Em tempo de epidemias procedia-se a uma averiguação da situação das ruas, das vielas e dos caminhos, assim mesmo as praças eram também tidas em consideração, tal como as fontes e locais de venda, como acontecia com as bancas dos vendedores de vários produtos. Proibia-se a presença de lixo no espaço público, como cisco e outras sujidades, animais mortos nos espaços urbanos, nomeadamente cães, porcos, gatos, bestas e “outras cousas mortas fedorentas”, que deviam ser retiradas pelos moradores e colocadas nos vazadouros públicos, enfatizando-se os maus odores existentes, a existência de fontes com vísceras, e responsabilizam-se os moradores pela limpeza das ruas (Araújo, 2022). Verificavam-se ainda os canos de água e comunicava-se à Câmara os perigos existentes. Apesar das medidas, as ruas não se apresentavam limpas nem asseadas.

As feiras, os mercados, as procissões eram igualmente proibidos, por aglomerarem muita população (Abreu, 2014), ao mesmo tempo que as portas das cidades eram fechadas, impedindo a circulação de fora para dentro e ao inverso. Para fazer desaparecer odores e purificar o ar, queimavam-se ervas em algumas ruas, de modo a contribuir para a saúde pública. Quem prevaricasse incorria numa coima ou mesmo em pena de prisão. Apesar da ameaça, o fato de as posturas serem reiteradas, prova que não eram cumpridas e que, passado o período mais crítico, as práticas antigas voltavam. Competia às Câmaras tomar medidas para combater as epidemias, de modo que as posturas se integravam num leque de outras medidas, que passavam desde logo pela nomeação de guardas-mores da saúde, encarregues de fazer cumprir o regimento de saúde local. Após notícia da existência de uma epidemia nas proximidades, as Câmaras tomavam medidas para que não passasse os muros da localidade, pese embora as dificuldades com que se defrontavam. Guardar as portas das cidades era fundamental para que a doença não entrasse, circulando através delas apenas os que estavam autorizados no regimento da saúde.

A não resposta da medicina sobre as causas das doenças, atribuindo-as à vontade divina, também sobre a cura, criava espaço para a manutenção e repetição de práticas populares ancestrais. Porém, o século XVIII é portador de algumas novidades em termos médicos. O surgimento da vacina contra a varíola constituiu um enorme avanço, pese embora a relutância de alguns na sua administração, que progressivamente se foi consensualizando. Essa doença tornou-se mais assídua no começo da Idade Moderna e fustigou-a até o surgimento da vacina. Converteu-se numa das enfermidades mais temidas, pela sua letalidade e ainda por transfigurar as pessoas ou mesmo causar-lhes a cegueira. Atacava primordialmente crianças, mas os adultos não escapavam. O desconhecimento era um fator de peso, ao que se juntava a falta de médicos e em muitos lugares a existência somente de cirurgiões, principalmente no interior do país, vários deles pouco informados sobre as novidades médicas, recorriam ao que conheciam e repetiam (Barreiros, 2014). Em Portugal, a inoculação fez-se quando já estava em curso noutros países, como a França, onde a temática foi objeto de um intenso debate no universo dos médicos, mas também na Suécia. A primeira obra do médico português Jacob de Castro Sarmento, escrita em inglês, foi dedicada a esta doença. A sua tradução para português e posterior divulgação acabou por ser muito relevante para a inoculação da vacina e o tratamento da doença (Pinto, 2021).

Em Setecentos defendia-se que a conservação da saúde dependia do estado da atmosfera, pelo que era necessário tratar de a depurar em situações de epidemia. Apesar das preocupações higienistas do século XVIII, que ditaram cuidados a adotar com o corpo, os espaços, os equipamentos, a tradição manteve-se, principalmente nos grupos populares, e só nos séculos seguintes se conheceram sérias mudanças. Porém, ainda em Setecentos, vários médicos portugueses adiantaram um conjunto de propostas tendentes a melhorar as condições de higiene e a preservar a saúde pública, de que se destaca Ribeiro Sanches, defensor de profundas mudanças na higiene e nos hospitais.

## Considerações finais

Analisar as epidemias no Portugal moderno é ainda um exercício incompleto, por não existirem estudos monográficos para todas as suas regiões, ficando-se, por isso, com ideias parcelares relativamente à sua cobertura geográfica. O mesmo não se pode dizer quanto às doenças, pois é muito claro que as conhecidas para o resto da Europa encontraram terreno fértil no nosso país para se propagarem, desorganizarem a vida das populações, amedrontarem e matarem.

A análise que efetuamos procurou sobretudo demonstrar como o meio ambiente influenciava a saúde pública e facilitava a instalação e propagação das doenças. Num período em que a produção estava totalmente dependente do clima, chuvas prolongadas ou fora do tempo, geadas e temporais ou falta de água tinham repercussões nas colheitas e estas na disponibilidade de grãos para consumo, o que fazia subir de imediato os preços dos produtos, e não somente dos cereais. A ascensão dependia da procura e da oferta, mas em alguns períodos foi proibitivo o acesso ao pão para as pessoas mais vulneráveis. Dependendo de cada época e até do lugar, a incidência desses fatores podia ser maior ou menor, mas as repercussões faziam-se sempre sentir, debilitando os mais fracos, enfraquecendo os corpos e expondo-os às doenças, o que, aliás, era facilitado por outras condições de falta de higiene e de insalubridade. A percepção que se tinha das doenças e a forma como eram entendidas, não potenciavam a alteração de comportamentos, por exemplo, no que tocava à limpeza do corpo, à lavagem e substituição da roupa, à forma como eram perspectivados o espaço público e a casa.

Os fatores analisados intervieram na incidência da doença junto da população, e a intensidade de cada uma delas era só por si suficiente para criar um ambiente de medo, mas não raramente várias surgiam associadas, aumentando o temor e o impacto da morte (Ruiz-Domènec, 2020).

Dependente do clima para se alimentar, a população assistia aos seus caprichos sem os entender, mas sabia que as suas consequências lhe podiam ser muito nefastas. A subida do preço dos cereais e a carestia alimentar, quando combinada com uma alimentação pouco diversificada e pobre, com água de pouca qualidade, com a falta de higiene corporal, ruas e praças com imundices, malcheirosas, infetas e com casas pouco asseadas e pobres resultavam em contextos convidativos ao surgimento de doenças, à sua propagação e ao aumento do medo e da morte.

A reflexão efetuada aponta para um Portugal muito debilitado em termos sanitários, quer particulares, quer públicos, com uma incidência grande de epidemias e uma forte resistência à mudança, pese embora algumas alterações surgidas em termos médicos e de higiene no século XVIII. Sinaliza ainda a relação existente entre meio ambiente e saúde pública, demonstrando o seu impacto nas epidemias.
